# Gut dysbiosis in mice genetically selected for low antibody production

**DOI:** 10.1186/s13099-017-0193-x

**Published:** 2017-08-07

**Authors:** Ana Carolina da Silva Santos, José Ricardo Jensen, Silvio Luis de Oliveira, Josias Rodrigues

**Affiliations:** 1 Department of Microbiology and Immunology, Institute of Biosciences of the State University of São Paulo (UNESP), Campus de Rubião Junior, Botucatu, SP 18618-961 Brazil; 2 Laboratory of Immunogenetics, Butantan Institute, Av. Dr. Vital Brazil 1500, São Paulo, SP 05503-900 Brazil

**Keywords:** Antibody, Prevotellaceae, Bacteroidetes, Ruminococcaceae, Firmicutes, Microbiome

## Abstract

**Background:**

Dysbiosis is linked to the cause of several human diseases, many of which having an immunity related component. This work investigated whether mice genetically selected for low or high antibody production display differences in intestinal bacterial communities, and consisted in the comparison of fecal 16SV6–V8 rDNA PCR amplicons resolved by temperature gradient gel electrophoresis (TGGE) of five each of low (LIII) and high (HIII) antibody producing mice. 16SV6 rDNA amplicons of 2 mice from each line were sequenced.

**Results:**

LIII mice were grouped in a single TGGE cluster, displayed a low α-diversity, and were distinguished by low Firmicutes/Bacteroidetes ratio.

**Conclusion:**

The results suggest that genetically driven low antibody production in mice is associated with gut dysbiosis.

## Background

Proper functioning of the immune system (IS) depends on its modulation by body associated microorganisms, in a process starting early after birth, lasting through all life time and which contributes for the shaping of microbial ecosystem itself, ultimately influencing body homeostasis. The importance of a fine-tuned microbiota-immunity balance can be illustrated by multiple human disorders having as an underlying basis defects in immune response and accompanying gut dysbiosis, such as autoimmune and inflammatory bowel diseases [1]. The use of animal models has been essential for the works unraveling IS functions and for understanding of immune-related diseases. Examples of such models include mice lineages bi-directionally selected for high and low quantitative antibody responses to a given antigen, but whose altered response extends to antigens unrelated to those employed in the selective process [2]. In addition to antibody production, these mice, which are represented by five selections obtained from an outbred Swiss mice foundation population (F0) [3], show interline differences with respect to other biological features. For example, selection III low antibody producer mice (LIII) display a higher resistance to chemically induced skin tumorigenesis [4] and to *Salmonella typhimurium* infection [5], higher susceptibility to pristane-induced arthritis (PIA) [6] and higher longevity following intraperitoneal injection of *Mycobacterium leprae* heat shock protein Hsp65 [7]. On the other hand, selection III high responder mice (HIII) are more resistant to *Trypanosoma cruzi* infection than animals of the LIII lineage, a feature which was shown to be gender-specific (females are more resistant than males) and correlated with interferon-γ and nitric oxide production by peritoneal lymph node cells [8]. The polarity with which these traits are manifested in between the lineages is the result of selective pressure towards the accumulation of quantitative trait loci endowed with opposite modulatory effects on the antibody biosynthesis pathway [9]. Given several examples of reciprocal influence of gut microbiota and immune system [10], we investigated whether immunity related and other interline differences between LIII and HIII could have an impact on fecal bacteriome of these mice lineages.

## Materials and methods

### Animals

Comprised 8–12 weeks-old female mice of Selection III, derived from the original outbred population [9], and raised at Butantan Institute, SP Brazil, in groups of five animals per cage, under the same temperature and supplied with the same kind and amount of feed and water. To check their immune responsiveness status, these animals are bi-monthly challenged with *S*. *typhimurium* flagellar antigen, and their corresponding Ab title determined by agglutinin assays.

Five each of low (LIII) and high (HIII) antibody producer lines were randomly taken for fecal bacteriome determination.

### DNA purification and PCR

The stools were submitted to DNA purification, using the QiaAMP DNA stool mini kit (Qiagen, Hilden, Germany) and the fecal DNA used as templates for PCRs carried out to amplify the sequences encoding the 16SV6–V8 rRNA (16SV6–V8 rDNA). The resulting 433 bp amplicons were resolved by temperature gradient gel electrophoresis (TGGE). PCR primers were U968 (GAACGCGAAGAACCTTAC) attached to a 40 bases GC clamp and L1401 (GCGTGTGTACAAGACCC). PCR conditions and components are described elsewhere [11]. Fecal DNAs of 4 (2 LIII and 2 HIII) randomly selected mice were submitted to additional PCRs to amplify the 16SV6 rRNA gene (16SV6 rDNA), whose amplicons were subsequently sequenced. PCR conditions and primers for 16SV6 rDNA were according to Andersson and co-workers [12]. The PCR was carried out in a 25 µL reaction mix, which included 1 µL (0.2 µM) of each primers, 0.5 µL DNA (20–50 ng) and 22 5 µL of platinum high fidelity supermix (Invitrogen, cod 12532016). PCR conditions were as follows: initial denaturation at 95 °C/5 min, followed by 30 cycles of 95 °C for 40 s (denaturation), 55 °C/40 s (annealing) and 72 °C/1 min (extension) plus a final extension of 72 °C/7 min. To eliminating by-products of the reaction, the PCR amplicon, a fragment of 330 bp, was purified by 2 successive mixing with the Agencourt AMPure X (Beckman Coulter, cod A63881) in a 1.5 mL Low bind polypropylene tube (Eppendorf, cod 22431021) and washing in 70% ethanol. After elution of the purified DNA in TE, the concentration of the amplicon was estimated with the Qubit 2.0 instrument, applying the Qubit dsDNA HS assay kit (Invitrogen, cod Q32851) and the Qubit 2.0 fluorometer (Invitrogen, cod Q32866).

### TGGE of 16S V6–V8 rDNA amplicons

16SV6–V8 rDNA amplicons were purified as described above and run in a 0.8 mm polyacrylamide (6% [wt/vol] acrylamide/biscrylamide [37.5:1], 8 M urea, 20% [vol/vol] formamide, 2% [vol/vol] glycerol, 0.09% [vol/vol], TEMED and 0.4% [vol/vol] ammonium persulfate) gel in a TGGE mini system (Biometra, Göttingen, Germany) for 2 h at 130 V, under a 40–44 °C temperature gradient, with 1×TAE as the running buffer. Following electrophoresis, the gel was fixed, silver stained according to the TGGE mini system manufacturer’s protocol, dried at room temperature and submitted to image capturing at the Gel doc EZ Imager (Biorad, Hercules, USA). Similarities or divergence among bacterial communities of distinct fecal samples were determined by pair-wise comparison of band profiles from the 16SV6–V8 rDNA amplicons TGGE. The relatedness among mice fecal bacteriome were represented in a dendrogram constructed with the help of DendroUPGMA [13].

### Clonal amplification and sequencing

Sequencing was preceded by clonal amplification, by an emulsion PCR, of the 16SV6 rDNA amplicons. To accomplish this, each of the four amplicons was diluted to a concentration of 1.56 × 10^8^ DNA molecules per microliter and then a pool was formed by mixing 5 µL of each of distinct amplicons. The pooled DNA was attached to the surface of ion sphere particles (ISPs) using the IonPGM™ Template OT2 400 kit (Life Technologies, cod 4480974) and the corresponding protocol (OT2 400 kit, Publication No. MAN0007219, Rev. 2.0). The emulsion PCR was carried out in the Ion OneTouch™ 2 System (Life Technologies), the quality of amplification was checked and ISP enrichment was performed in the Ion OneTouch™ Enrichment System (Life Technologies). Sequencing primers were then annealed to the ISPs’ single stranded DNA, following Ion PGM sequencing 400 kit protocol (Life Technologies Publication No. MAN0007242). Sequencing proceeded in the Ion torrent personal genome machine (PGM™, Life Technologies) in a ISP loaded 314™ chip. Low quality and polyclonal sequence reads, as well as primers and barcodes were filtered out of the data and the 16SV6 rDNA sequences were available as a FastQ file.

### Bioinformatics and statistical analyses

Quality filtered FastQ files were uploaded to MG-RAST server [14], where reads from mouse origin were removed and unique bacterial sequences classified at distinct taxonomic levels, using Ribosomal Database Project [15] as a reference. Clustering of reads in operational taxonomic units (OTUs) was based on an identity threshold of 97%, e-value cutoff of 1e–5, and minimum alignment cutoff of 15. OTUs were used for α-diversity calculation, rarefaction curve and heatmap construction and principal component analyses (PCoA). These analyses were performed with the Bioinformatics tools at the MG-RAST website (http://metagenomics.anl.gov/).

## Results

**Fig. 1:**
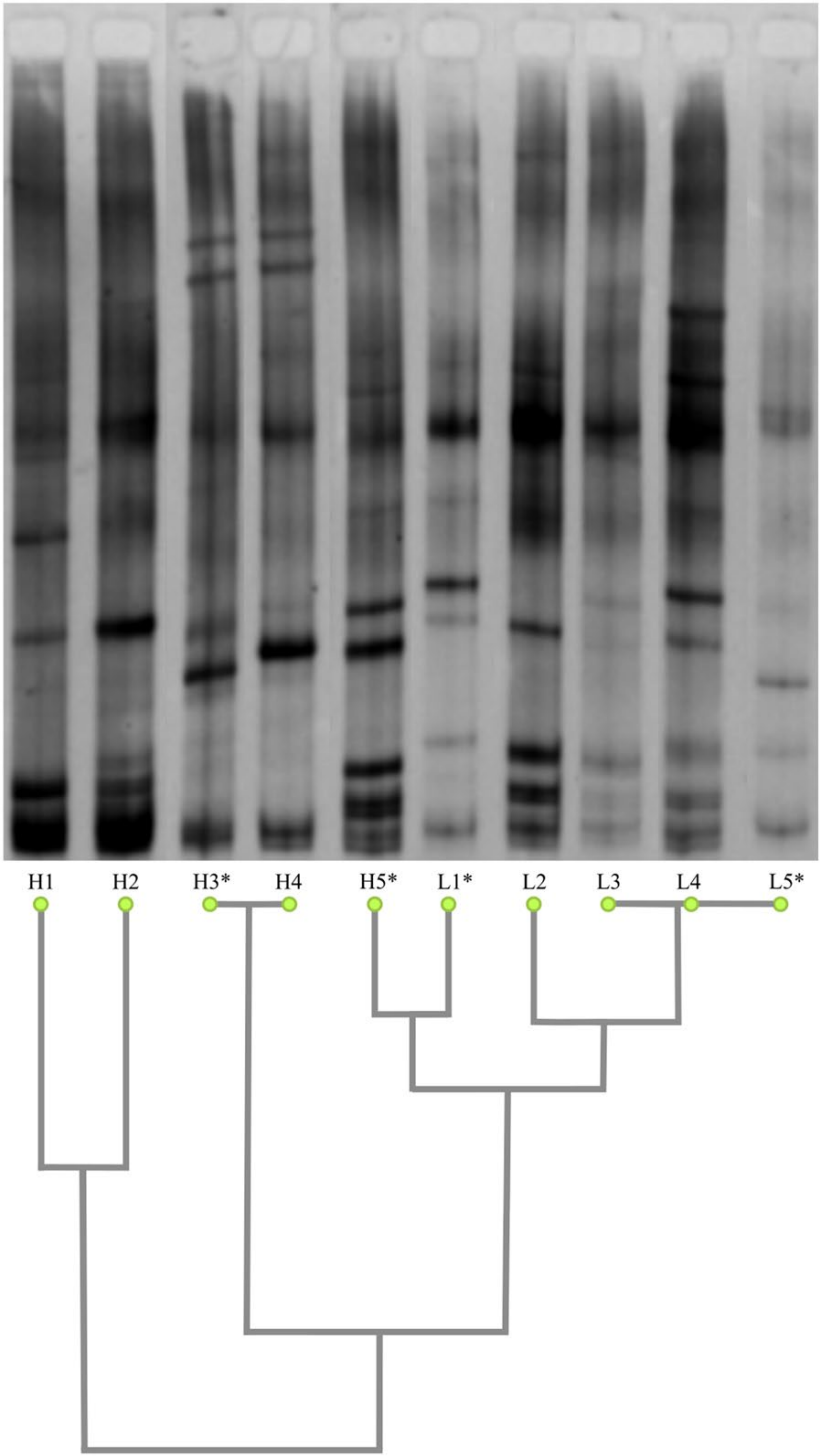
Band profiles resulting from the separation, by TGGE, of 16SV6–V8 amplicons of LIII and HIII mice’s fecal DNA and clustering of the samples based on the corresponding bacterial diversity. Dendrogram was constructed using *d*_*G*_ matrix data in DendroUPGMA web utility with Pearson correlation coefficient

**Fig. 2:**
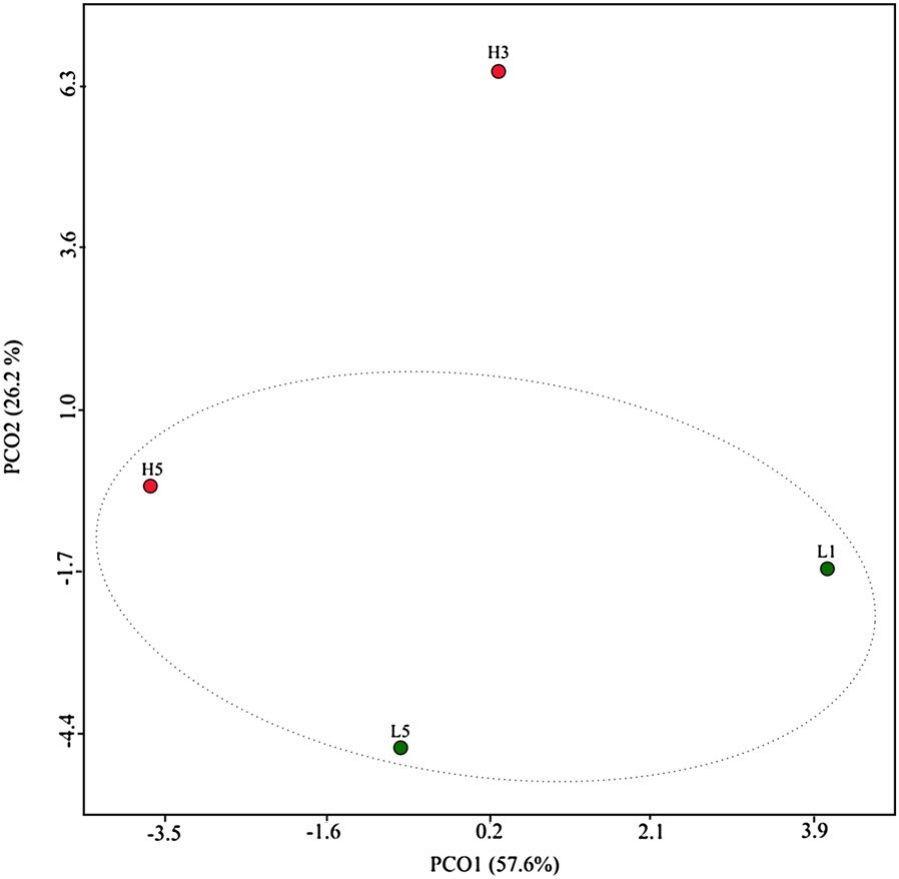
PCo relating each of the four mice’s fecal bacteriomes

Comparative analysis of fingerprints generated by TGGE of 16SV6–V8 amplicons of the fecal DNA enabled the grouping of all LIII mice, but not of HIII, in a single cluster (Fig. 1). There was an extensive variation among HIII mice’s fingerprint, one of which (H5) clustered with LIII. Fingerprints from two mice of the HIII (H3 and H4) and three from LIII (L3–L5) lines were similar or identical. Fecal DNA from two mice of each line (H3, H5, L1 and L5, marked with asterisk in Fig. 1) had been randomly selected for 16SV6 rDNA amplification and sequencing, aiming at bacterial identification, prior to the TGGE experiment. PCo based on 16SV6 rDNA sequencing data reflecting bacterial abundance and diversity present in the four mice’s stools grouped H5 with LIII mice (Fig. 2), a result concordant with that of TGGE.

**Fig. 3:**
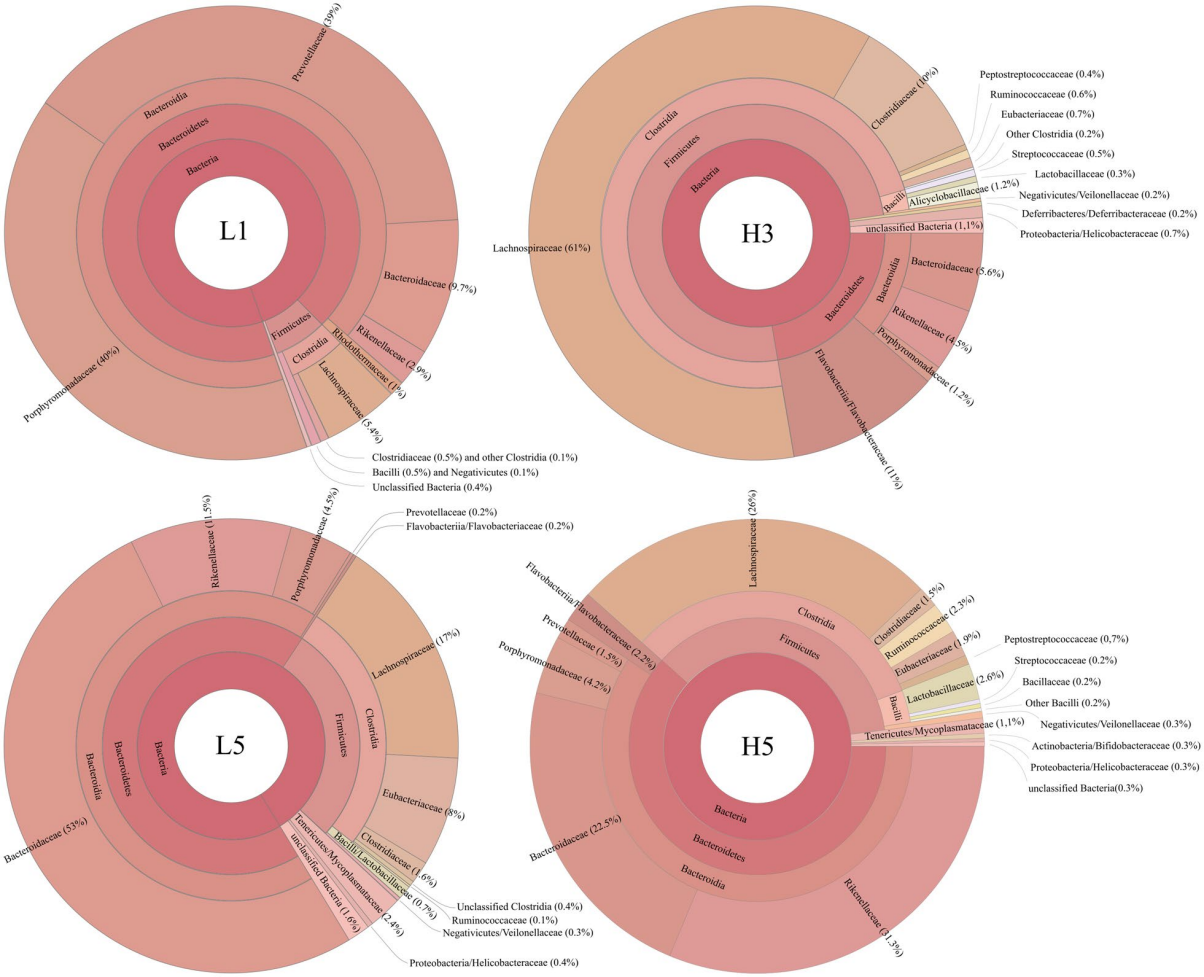
Chart displaying relative abundance of bacterial taxa identified in each mouse’s fecal bacteriome

Average alfa diversity for HIII and LIII was 8.2 ± 2.1 and 6.2 ± 1.9, respectively, with at least 97% of the 16SV6 rDNA reads in both HIII and LIII mice corresponding to Firmicutes (F) and Bacteroidetes (B) (Fig. 3). F/B ratios for L1, L5, H3, and H5 were 0.07, 0.4, 3.4, and 0.6, respectively. Given the identity of TGGE profiles between some sequenced and non-sequenced samples (H3 and H4, L3–L5, Fig. 1) one could extrapolate the data from sequencing for samples displaying identical TGGE band profiles. Therefore, assuming that TGGE of 16S V6–V8 rDNA amplicons at least partially reflect sequencing derived data as well as the existence of identical (L3–L5) or similar (L2 to L3–L5) TGGE band profiles in the LIII cluster, low F/B ratios denoting a higher prevalence of Bacteroidetes could be a marker of this line’s fecal bacteriome. Furthermore, Bacteroidetes in this cluster was represented mainly by the class Bacteroidia (Over 99% of Bacteroidetes in LIII and 96.4% in H5 mouse), which nonetheless corresponded to different family in distinct samples (high abundance of Prevotellaceae and Porphyromonadaceae in L1, Bacteroidaceae in L5, and Bacteroidaceae and Rikenellaceae in H5) (Fig. 3). Of the four mice whose fecal 16SV6 rDNAs were sequenced, the most distinct bacteriome pattern was found in H3, characterized by a higher abundance of Firmicutes (75%), the greater part of which corresponded to Lachnospiraceae. In addition, in this mouse’s stool, Bacteroidia was less represented than in those of the other mice analyzed and half of Bacteroidetes corresponded to Flavobacteriia (Fig. 3). Since H3 shared a TGGE profile with H4, these mice may have displayed similar or identical bacteriomes, which, in addition to the superior abundance of Firmicutes, include a higher prevalence of Ruminococcaceae and lower prevalence of Prevotellaceae (Fig. 3).

Comparative analysis, at the species level, of the sequencing data from the two mice of each lineage, taking in account only the most abundant Bacteria (arbitrarily considered as those found with a minimum of 100 reads in at least one of the mice’s bacteriomes), significant interline variation was seen only at the higher prevalence of *Elizabethkingia meningoseptica* (formely *Chryseobacterium meningosepticum* and *C. miricola,* Flavobacteriaceae) and *Clostridium hathewayi* (Clostridiaceae) in HIII (*E. meningoseptica*: 698 ± 44 in HIII × 38 ± 30 reads in LIII, p = 0.003; *C. hathewayi*: 198 ± 58 in HIII ×12 ± 1 reads in LIII, p = 0.04). Although 43 and 54 distinct species were found exclusively in LIII and HIII lines respectively, only the particular association of *Ruminococcus torques* (41 ± 14 reads) with HIII mice was statistically significant (p = 0.03).

## Discussion

The present study aimed at investigating whether genetically accumulated differences in the ability to produce antibody could influence the structure of mice’s intestinal bacteriome. This was performed by the analysis of stools from Biozzi mice, derived from Swiss lineage by successive inbreeding to display differential quantitative response to *S. typhimurium* antigen (selection III) [9]. TGGE of 16SV6-V8 rDNA amplicons of fecal DNA from an equal number (five) of each low (LIII) and high (HIII) antibody producer mice revealed a higher inter-individual variation in bacteriomes of the later, an observation also drawn from 16SV6 rDNA sequence analyses of fecal samples from two mice of each line. The amplitude of variation among HIII mice were so high that one of the HIII mice (H5) carried a bacterial community more similar to the LIII mice’s bacteriome than to that of its line. LIII mice’s fecal bacteria were enriched in Bacteroidetes of the families Prevotellaceae, Porphyromonadaceae, and Bacteroidaceae. Bacteroidetes in HIII mice included Flavobacteriaceae, which was absent in LIII. Firmicutes, represented mainly by Lachnospiraceae, was dominant in one of the HIII mice’s bacteriome. Since TGGE profile of this mouse’s fecal DNA was identical to that of other mouse of the same line whose DNA was not sequenced (Fig. 1), it is very likely that both of these mice carried the same bacteriome, marked by a Firmicutes–Lachnospiraceae higher abundance. Although not all mice’s fecal 16S rDNA have been sequenced, the fingerprints identity of some sequenced and non-sequenced samples strongly suggests the similarity of their fecal bacteriome, given a good association between TGGE and sequencing data already demonstrated elsewhere [16]. Therefore, based on this assumption, results of sequencing of L1 and L5 samples could be extrapolated for at least two other mice of the LIII line, which displayed similar or identical fingerprints to theirs, but whose samples were not sequenced. As the sequencing data of the two LIII mice’s samples revealed dysbiosis, this could be considered a feature of LIII mice’s bacteriome. However, this assumption could not be valid for the HIII mice which was shown to be extremely variable, in regard to their fecal bacteriome, an observation which can be drawn not only from TGGE data but also from sequencing, wherein the two HIII samples was shown to bear divergent bacterial communities (Figs. 2, 3).

Lachnospiraceae and Ruminococcaceae, primary butyrate producers in human gut, have been negatively associated with human diarreal disease [17]. In the case of Lachnospiraceae, a negative correlation has also been shown with disease activity in IL-10 (−/−) murine model of colitis [18]. *Ruminococcus torques* (Ruminococcaceae), shown here as an eventual microbiota signature of the HIII line, is less abundant in CD patients, particularly those displaying a C-reactive protein positive reaction [19]. *Clostridium hathewayi,* along with other butyrate producing Clostridia have been demonstrated to induce the proliferation of T-regulatory (Treg) cells and to attenuate symptoms in experimental colitis and allergic diarrhea when administrated orally to mice [20]. All of the above correspond to examples of bacteria which were found to be enriched in HIII mice and associated with anti-inflammatory properties and health conditions.

LIII and HIII mice are respectively susceptible and resistant to PIA, and susceptible animals show a higher number of pro-inflammatory cytokines IL-1β, IL-12 and TNF-α producing splenocytes [6]. In cyclophosphamide-treated C57BL/6, a repression in the production of IL-12 and TNF-α with an impact on bacterial composition of fecal bacteriome has been observed. The alterations in these mice’s bacteriome consisted in the rise in the abundance of Lachnospiraceae and reduction in that of Prevotellaceae [21]. Since in the present study these two families were abundant respectively in HIII and LIII mice, the known higher inflammatory activity seen in the later seems to support the association of fecal enrichment of Prevotellaceae and low abundance of Lachnospiraceae with inflammation. Indeed, over-representation of Prevotellaceae has also been seen in the gut microbiota of mice bearing colonic epithelial cells deficient in the expression of the NLRP6 inflammasome [22], and in gut biopsies of ulcerative colitis patients [23]. Therefore, accumulated traits resulting from the genetic selection process in LIII mice might have affected some microbial recognition function leading to an aberrant host-microbial cross-talk, and hence dysbiosis.

Although the high inter-individual variation among HIII mice, deduced from TGGE fingerprints, could mean lack of a particular feature in this line’s bacteriome, two of them (H3 and its similar fingerprint displaying H4 line’s match) were shown to possess a higher diversity and displaying a taxa composition which, in the light of available literature information, could approximate to a non-disease favoring environment in the gut. Opposing to this normobiosis picture, the less heterogeneous LIII mice’s bacteriome included bacterial taxa associated with a pro-inflammatory dysbiosis condition. These observations should nonetheless be validated by future studies, with the investigation of higher number of animals as well as taking in account other variables such as cage effect [24], to minimize inter-individual variation.
